# *In vivo* efficacy of cefiderocol against ceftolozane/tazobactam- and ceftazidime/avibactam-resistant *Pseudomonas aeruginosa* carrying the AmpC E219K substitution

**DOI:** 10.1128/aac.00724-26

**Published:** 2026-09-16

**Authors:** Arianna Rodríguez-Coello, Michelle Outeda-García, Andrea García-Pose, Lucía Gonzalez-Pinto, Gabriela Alejandra Báez-Barroso, Paula Guijarro-Sánchez, Andrea Muras, Biel Taltavull, Pablo Fraile-Ribot, Isaac Alonso-Garcia, Antonio Oliver, Juan Carlos Vázquez-Ucha, Jorge Arca-Suárez, Germán Bou, Alejandro Beceiro

**Affiliations:** 1 Servicio de Microbiología and Instituto de Investigación Biomédica A Coruña (INIBIC), Complexo Hospitalario Universitario A Coruña 16811, A Coruña, Spain; 2 CIBER de Enfermedades Infecciosas (CIBERINFEC), Instituto de Salud Carlos III 38176, Madrid, Spain; 3 Servicio de Microbiología Hospital Universitario Son Espases Instituto de Investigación Sanitaria Illes Balears (IdISBa) 219656 , Palma de Mallorca, Spain; 4 Departamento de Fisioterapia, Medicina y Ciencias Biomédicas, Universidad de A Coruña 16737 , A Coruña, Spain; University of Pennsylvania Perelman School of Medicine, Philadelphia, Pennsylvania, USA

**Keywords:** *Pseudomonas aeruginosa*, cefiderocol, ceftolozane/tazobactam, ceftazidime/avibactam, PDC, E219K, murine infection

## Abstract

Chromosomal AmpC variants selected during ceftolozane/tazobactam or ceftazidime/avibactam therapy may affect cefiderocol activity. We evaluated the impact of the E219K substitution on cefiderocol efficacy using isogenic *Pseudomonas aeruginosa* pairs in a murine bacteremia model. Cefiderocol achieved greater bacterial reductions in parental isolates than in E219K derivatives, despite susceptible MICs. Cefiderocol failed to achieve stasis against a cefiderocol-resistant isolate. E219K impaired cefiderocol efficacy *in vivo*, supporting close monitoring of cefiderocol activity against isolates carrying this variant.

## INTRODUCTION

Treatment of infections caused by *Pseudomonas aeruginosa* is increasingly challenging because of limited antipseudomonal options and its ability to develop resistance via chromosomal mutations. Mechanisms, such as AmpC (*Pseudomonas*-derived cephalosporinase, PDC) hyperproduction, efflux overexpression, and OprD porin loss, generate difficult-to-treat phenotypes, further compounded by MDR/XDR high-risk clones with transferable β-lactamases. Recently introduced β-lactam/β-lactamase inhibitor combinations, such as ceftolozane/tazobactam and ceftazidime/avibactam, represent a major advance in therapy; however, resistance may rapidly emerge, frequently driven by amino acid substitutions in critical regions of the intrinsic AmpC enzyme (1–5).

Cefiderocol is a novel cephalosporin with enhanced stability against β-lactamases and antibacterial activity. Its chlorocatechol moiety enables iron binding and siderophore-mediated uptake. In *P. aeruginosa*, mutations affecting iron uptake systems, including *piuC* or *pirA*, and production of specific β-lactamases with high cephalosporinase activity, such as OXA-15, NDM, or PER, may increase cefiderocol MICs (6–9). Notably, cefiderocol often retains activity against *P. aeruginosa* isolates harboring PDC β-lactamase substitutions conferring resistance to ceftolozane/tazobactam or ceftazidime/avibactam, including strains previously exposed to these combinations (5, 10), supporting its potential as a therapeutic alternative in case of resistance development. However, previous studies have identified the E219K substitution as a frequently selected PDC variant after ceftolozane/tazobactam or ceftazidime/avibactam therapy, with an impact on cefiderocol activity (11). Although these isolates often remain susceptible to cefiderocol, they exhibited a significant increase in MIC compared with their parental isolates, as demonstrated by our group and others using recombinant strains and steady-state kinetics (1, 3–5, 12–16). Functional and structural data indicate that E219K enhances cefiderocol hydrolysis by improving substrate accommodation within the PDC active site, consistent with increased hydrolytic efficiency (1, 5).

These observations raise important questions about the efficacy of cefiderocol in the clinical challenge posed by the continuing emergence of ceftolozane/tazobactam- and ceftazidime/avibactam-resistant *P. aeruginosa* strains. Thus, we aimed to evaluate the impact of the E219K substitution on the therapeutic efficacy of cefiderocol using a murine bacteremia model. The study hypothesized that the amino acid substitution in this key residue may partially compromise cefiderocol activity *in vivo*, potentially limiting its clinical utility against infections caused by PDC E219K-producing strains.

Two pairs of isogenic *P. aeruginosa* clinical isolates were included: pre-treatment susceptible isolates and their corresponding post-treatment derivatives, the latter selected during ceftolozane/tazobactam therapy and resistant to both ceftolozane/tazobactam and ceftazidime/avibactam (101-E5/103-H8 and 5A-HUSE/5B-HUSE). Both pairs were characterized at Son Espases Hospital (Palma de Mallorca, Spain) as part of previous studies (3, 13). In addition, the isolate 11-CHUAC from A Coruña Hospital (Coruña, Spain) was also included and characterized by exhibiting a high MIC for cefiderocol (16 mg/L) (11, 17).

All isolates were characterized by whole-genome sequencing (WGS) in previous studies using Illumina technology and analyzed using the *Pseudomonas aeruginosa* resistome explorer (PaREx) (18). Antimicrobial susceptibility testing was performed as described in the Supplementary Material section. The susceptibility profiles, duration of ceftolozane/tazobactam treatments, and β-lactam resistance mechanisms of these isolates are summarized in Table 1.

**TABLE 1:** Microbiological and clinical features of the *P. aeruginosa* isolates used to evaluate the impact of the PDC E219K substitution on cefiderocol activity

*P. aeruginosa* isolate ID	PDC variant	ST	CAZ (*R* > 8)^*a*^	CAZ/AVI^*b*^ (*R* > 8)	TOL/TAZ (*R* > 4)	FDC (*R* > 2)	IMI (*R* > 4)	IMI/ REL (*R* > 2)	β-Lactam resistance mechanisms	Clinical context/days of therapy with ceftolozane/tazobactam/sample	Reference
101-E5	PDC-1	175	16	4	2	0.25	16	2	OprD Q142stop AmpR G154R	Pre-treatment isolate/NA/bronchial aspirate	(3, 19)
103-H8	PDC-221 (E219K)	175	64	32	32	1	4	1	OprD Q142stop AmpR G154R AmpC E219K	Post-treatment isolate/10/bronchial aspirate	(3)
5A-HUSE	PDC-533	175	64	8	2	0.06	32	4	OprD Q142stop AmpR G154R	Pre-treatment isolate/NA/bronchial aspirate	(13)
5B-HUSE	PDC-709 (E219K)	175	64	64	16	0.5	2	2	OprD Q142stop AmpR G154R AmpC E219K	Post-treatment isolate/14/bronchial aspirate	(13)
11-CHUAC	PDC-221 (E219K)	3611	≥512	256	≥512	16	4	1	PBP3 R504C OprD S57fs^*c*^ MexR T32fs AmpC E219K	Post-treatment isolate/14/tracheal aspirate	(11, 17)

^*a*^ EUCAST v14.0 clinical breakpoints indicated.

^*b*^ Avibactam, tazobactam, and relebactam were fixed at 4 mg/L. CAZ, ceftazidime; CAZ/AVI, ceftazidime/avibactam; TOL/TAZ, ceftolozane/tazobactam; FDC, cefiderocol; IMI, imipenem; IMI/REL, imipenem/relebactam.

^*c*^ Frameshift (fs).

An experimental model of sepsis in immunocompetent mice was used to evaluate the efficacy of cefiderocol in systemic infections by the aforementioned strains. Nine- to twelve-week-old male BALB/c mice were used, distributed in groups of 9–12. Mice were infected with the previously determined LD_100_ of each isolate. Cefiderocol treatment was administered according to the humanized dosing regimen described by Ghazi et al. (20). To reproduce human plasma exposures achieved with cefiderocol (2 g every 8 h as a 3-h infusion), mice received sequential doses of 15, 20, 25, 10, and 5 mg/kg at 0, 1, 2, 4, and 6 h, respectively, every 8 h. The applicability of the cefiderocol dosing regimen was preliminarily verified by measuring serum cefiderocol concentrations using bioassays with *Escherichia coli* ATCC 25922 and *Stenotrophomonas maltophilia* ATCC 13637 (21).

Treatments started 2 h after the onset of infection. Untreated control groups were included (inoculated with saline solution as vehicle control). Also, groups of four mice were inoculated with LD_100_ of each isolate and euthanized at 2 h (before the beginning of the treatment) to obtain the baseline of bacterial loads. Mice were treated and monitored for 24 h, euthanized with an overdose of thiopental sodium, and processed immediately after death. Bacterial density (log₁₀ CFU/g) was quantified in the spleens, lungs, and livers as previously described (22). Statistical differences between cefiderocol-treated and untreated groups were determined using Student’s *t* test.

*In vitro* susceptibility testing showed that the isolates selected after the ceftolozane/tazobactam treatments, 103-H8 and 5B-HUSE, had higher MICs to ceftazidime/avibactam, ceftolozane/tazobactam, and cefiderocol than their parental isolates, although the increase was more modest for cefiderocol. Conversely, imipenem MICs decreased (Table 1). WGS analysis previously performed showed that the post-treatment isolates differed from their pre-treatment counterparts exclusively by the single amino acid substitution E219K in the parental PDC β-lactamase (PDC-1 for 101-E5 and PDC-533 for 5A-HUSE).

It should also be noted that the strains included in this study have a genetic background associated with increased *ampC* expression. The two pairs 101-E5/103-H8 and 5A-HUSE/5B-HUSE carried the AmpR G154R substitution, an amino acid change frequently found in clinical strains belonging to the ST175 clone and previously characterized for its ability to increase *ampC* expression (23). Thus, in a previous study, the strains of the 101-E5/103-H8 pair showed *ampC* expression levels 554- and 391-fold higher, respectively, than those of PAO1 (19). Regarding the 11-CHUAC clinical isolate exhibiting a cefiderocol MIC of 16 mg/L, well above the clinical breakpoint, it displayed the PDC E219K substitution and showed 12-fold higher *ampC* expression than PAO1, along with alterations in PBP3 and MexR inactivation (11, 17). These susceptibility results are consistent with previous reports showing that E219K selected in *P. aeruginosa* treatment-emergent mutants under ceftolozane/tazobactam exposure significantly increases PDC catalytic efficiency (*k*_cat_/*K*_M_) toward cephalosporin substrates (1, 5).

**Fig 1:**
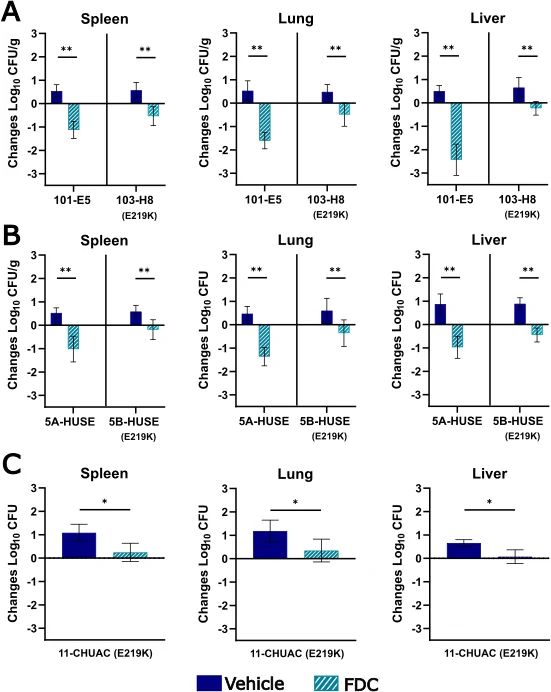
*In vivo* change in log_10_ CFU/g in three different organs of BALB/c mice (*n* = 9–12) infected intraperitoneally with *P. aeruginosa* strains after 24 h of treatment with cefiderocol and non-treated control. The solid line marks initial bacterial load (baseline). (**A**) Pre-treatment 101-E5 and post-treatment 103-H8 isogenic isolates; (**B**) pre-treatment 5A-HUSE and post-treatment 5B-HUSE isogenic isolates; and (**C**) 11-CHUAC isolate. Statistical analysis was performed by a Student’s *t* test, *P < 0.05, **P < 0.01.

In the murine infection models, cefiderocol showed significantly lower *in vivo* efficacy against E219K mutants than against their corresponding pre-treatment isolates, supporting a reduced therapeutic efficacy associated with this PDC substitution. In the parental isolate 101-E5, the differences in bacterial load between the untreated and cefiderocol-treated groups were 1.67 log₁₀ CFU/g in the spleen, 2.14 log₁₀ in the lung, and 2.94 log₁₀ in the liver (relative to the 2 h post-inoculation baseline). In contrast, cefiderocol exhibited reduced efficacy against the post-treatment isolate 103-H8 compared with the untreated group, achieving reductions of 1.11, 0.97, and 0.88 log₁₀ CFU/g in the spleen, lung, and liver, respectively (Fig. 1A; Table S1).

Regarding the second *P. aeruginosa* pair, the differences between the bacterial load of the pre-treatment isolate 5A-HUSE in the cefiderocol-treated group compared with the vehicle-treated group were 1.55, 1.84, and 1.85 log₁₀ CFU/g in the spleen, lung, and liver, respectively (Fig. 1B; Table S1). In contrast, in the post-treatment isolate 5B-HUSE, cefiderocol treatment displayed a reduction of bacterial load of 0.78, 0.97, and 1.39 log₁₀ CFU/g in the spleen, lung, and liver, respectively, again demonstrating decreased efficacy of cefiderocol against the post-treatment isolate harboring the PDC E219K substitution. The average net bacterial loads of these treatments are shown in Fig. S1A and B and Table S1. Direct comparison of cefiderocol efficacy between each pre-treatment/post-treatment isogenic pair showed significantly lower bacterial load reductions for the E219K-containing post-treatment isolates in five of the six organ comparisons. The only exception was the spleen of the 101-E5/103-H8 pair, where a similar trend was observed, with the difference narrowly missing the conventional threshold for statistical significance (*P* = 0.085).

Finally, against the 11-CHUAC isolate with high-level cefiderocol resistance, cefiderocol failed to achieve stasis at 24 h (Fig. S1C, Table S1). Relative to untreated controls, cefiderocol achieved only 0.84, 0.83, and 0.59 log₁₀ CFU/g reductions across the three organs, highlighting the markedly reduced efficacy of cefiderocol against a high-MIC strain (Fig. 1C). It should be noted that the failure to achieve stasis against this isolate cannot be attributed to E219K alone, as this isolate also exhibited *ampC* overexpression, PBP3 alterations, and MexR inactivation, which likely contributed collectively to the cefiderocol-resistant phenotype. Overall, these findings show that the AmpC E219K substitution reduces cefiderocol efficacy in a *P. aeruginosa* bacteremia model, as evidenced by higher bacterial counts in target organs, even when MICs remain below clinical breakpoints.

Most pharmacodynamic studies define efficacy using stasis (i.e., bacteriostatic effect) and 1–2 log₁₀ CFU reductions at 24 h. Regulatory agencies (e.g., European Medicines Agency [EMA] and U.S. Food and Drug Administration [FDA]) emphasize maximal bacterial reductions, balancing efficacy, toxicity, and cost. However, antibiotics with slower activity or higher MICs often fail to achieve ≥2 log₁₀ CFU/g reductions, and preclinical development, therefore, accepts stasis thresholds for less severe infections and ≥1 log₁₀ reductions for severe infections, such as pneumonia or bacteremia (24, 25). Taking these criteria into account, although the MIC values of cefiderocol in both post-treatment isolates remained below the clinical breakpoint for resistance (*R* > 2 mg/L), the results of our *in vivo* models suggest a notable reduction in efficacy. While the parental isolates reduced their bacterial load relative to the baseline, a mean of 1.55–2.94 log₁₀ CFU/g, the post-treatment strains reduced their load by only 0.78–1.39 log₁₀ CFU/g.

The strain-specific inocula used to achieve the previously determined LD100 values are shown in Table S2. The table also indicates the baseline bacterial burdens measured in the lungs 2 h after infection, immediately before treatment initiation (ranged from 6.8 to 7.4 log10 CFU/g of the lung). These baseline burdens are consistent with those commonly reported in antimicrobial PK/PD infection models. Previous studies indicate that bacterial burdens at treatment initiation are generally approximately 6–7 log10 CFU in the target tissue (26, 27). Given these relatively high baseline bacterial burdens, the potential influence of inoculum size on the effect of E219K should also be considered. A high bacterial burden could enhance the impact of E219K by increasing the total amount of PDC at the infection site and, consequently, cefiderocol hydrolysis. However, further studies using low- and high-inoculum conditions are needed to determine whether the effect of E219K on cefiderocol efficacy is inoculum dependent.

Previous murine models with extended-spectrum β-lactamase (ESBL) strains of *Klebsiella pneumoniae* and *Escherichia coli* have shown that increased MICs of cefepime or meropenem reduced the PK/PD parameter %T > MIC, and, therefore, their efficacy of the dosage regimen was not modified. Accordingly, a standard dose of cefepime against ESBL *E. coli* achieved only a reduction of 0.5–0.8 log₁₀ CFU/g (28), while meropenem achieved only a reduction of 0.5–0.9 log₁₀ CFU/g (29).

In the PERSEUS multicenter survey, cefiderocol showed >80% clinical effectiveness against severe *P. aeruginosa* infections, supporting its role as a key antipseudomonal. However, despite the high resistance rates to ceftolozane/tazobactam and ceftazidime/avibactam mainly driven by metallo-β-lactamases, the prevalence and clinical impact of E219K in cefiderocol-treated infections remain unclear (30). Our model indicates that the E219K Ω-loop variant can impair cefiderocol therapeutic efficacy despite MICs remaining within the susceptible range, supporting close monitoring, particularly after prior cephalosporin/β-lactamase inhibitor exposure. Although E219K alone may not exceed breakpoints, additional mechanisms, such as iron uptake alterations or PBP3 changes, may contribute to clinically relevant resistance, as observed in isolate 11-CHUAC and in our recent studies (4, 31). Nevertheless, these experimental findings do not demonstrate that the standard cefiderocol dosing regimen will necessarily result in clinical failure. Instead, this substitution may represent a risk factor for reduced efficacy, particularly in high-burden infections, when cefiderocol MICs approach the susceptibility breakpoint or when additional resistance mechanisms are present. These findings further suggest that E219K may act as an evolutionary intermediate toward higher-level cefiderocol resistance.

Another key PDC residue, L293P, was previously identified by our group after *in vitro* evolution with cefiderocol and associated with decreased susceptibility through increased affinity and enhanced hydrolysis (4, 5, 8). This substitution has also been detected in Enterobacterales exposed to cefepime (32). Beyond E219K and L293P, most PDC amino acid replacements in the AmpC, such as G156D or F121L, barely affect cefiderocol MICs compared with the wild-type PDC-1 enzyme, suggesting a limited contribution to cefiderocol resistance (4, 5).

These findings also suggest that genomic information can complement the interpretation of cefiderocol susceptibility based on MIC in selected contexts. In particular, the detection of substitutions in PDC, such as E219K, can identify isolates in which cefiderocol activity may be compromised despite MIC values falling within the susceptibility range. However, it should be noted that WGS should not replace phenotypic susceptibility testing, as cefiderocol activity may also be influenced by *ampC* expression, defects in iron uptake, alterations in PBPs, or changes in permeability and efflux. Genomic analysis can provide added value, especially in those *P. aeruginosa* isolates previously exposed to ceftolozane/tazobactam or ceftazidime/avibactam.

The emergence of PDC substitutions such as E219K may compromise the efficacy of cefiderocol and poses a serious clinical challenge. Since cephalosporin/β-lactamase inhibitor combinations lose effectiveness, and these AmpC variants affect cefiderocol, therapeutic options for multidrug-resistant *P. aeruginosa* are drastically limited: (i) if isolates with E219K substitutions maintain susceptibility to cefiderocol but have reduced *in vivo* efficacy, clinicians should carefully reassess cefiderocol use and ensure PK/PD-optimized dosing; (ii) combining cefiderocol with other β-lactamase inhibitors, such as xeruborbactam, currently under evaluation in a phase clinical trials (31, 33) may provide additional coverage against these enzymatic targets; and (iii) considering carbapenem/β-lactamase inhibitor combinations, such as imipenem/relebactam, as therapeutic options given the reduced potency of these PDC E219K variants.

In conclusion, while additional *in vitro* and *in vivo* studies, as well as clinical experience, are needed to decide the most suitable therapeutic strategy against *P. aeruginosa* strains carrying PDC variants, these data call for close monitoring the activity and effectiveness of cefiderocol against ceftolozane/tazobactam and ceftazidime/avibactam-resistant *P. aeruginosa*, particularly strains that have developed resistance through the E219K change in the Ω-loop.

## Data Availability

The raw sequence data of 101-E5/103-H8 and 5A-HUSE/5B-HUSE have been deposited in the European Nucleotide Archive under project number PRJEB113245, while data for 11-CHUAC have been deposited in the National Center for Biotechnology Information’s Sequence Read Archive under BioProject no. PRJEB79019.

## SUPPLEMENTAL MATERIAL

Supplemental material aac.00724-26-s0001.docx Fig. S1; Tables S1 and S2; Supplemental methods.

ASM does not own the copyrights to Supplemental Material that may be linked to, or accessed through, an article. The authors have granted ASM a non-exclusive, world-wide license to publish the Supplemental Material files. Please contact the corresponding author directly for reuse.
