# Supplementary material for: In vivo efficacy of cefiderocol against ceftolozane/tazobactam- and ceftazidime/avibactam-resistant Pseudomonas aeruginosa carrying the AmpC E219K substitution
Source: Antimicrob Agents Chemother. 2026 Sep 16;70(10):e00724-26. doi: 10.1128/aac.00724-26 (PMC13689382; doi:10.1128/aac.00724-26)
Supplement: Supplementary file 1 [file aac.00724-26-s0001.docx]

**SUPPLEMENTAL MATERIAL**

**TITLE**

***In vivo* efficacy of cefiderocol against ceftolozane/tazobactam- and ceftazidime/avibactam-resistant *Pseudomonas* aeruginosa carrying the AmpC E219K substitution.**

AUTHORS:

Arianna Rodríguez-Coello, Michelle Outeda-García, Andrea García-Pose, Lucia González-Pinto, Gabriela Alejandra Báez-Barroso, Paula Guijarro-Sánchez, Andrea Muras, Biel Taltavull, Pablo Fraile-Ribot, Isaac Alonso-Garcia, Antonio Oliver, Juan Carlos Vázquez-Ucha, Jorge Arca-Suárez, Germán Bou, Alejandro Beceiro.

**Supplementary Figure 1_______________________________Page 2**

**Supplementary Table 1________________________________Page 3**

**Supplementary Table 2________________________________Page 4**

**Supplementary material and methods___________________ Page 5**

**Supplementary Figure 1.** Net bacterial burden (Log_10_ CFU/g organ) after 24 h of treatment with cefiderocol in BALB/c mice (*n* = 9-12) infected intraperitoneally with *P. aeruginosa* strains. The bacterial load in spleen, lung and liver of the saline solution-treated groups (circle) versus those treated with cefiderocol (triangles) was determined for each strain. A) Pre-treatment 101-E5 and post-treatment 103-H8 isogenic isolates; B) pre-treatment 5A-HUSE and post-treatment 5B-HUSE isogenic isolates; and C) 11-CHUAC isolate. Statistical analysis was performed by a Student's *t* test, **P* < 0.05, ***P* < 0.01.

**Supplementary Table 1.** Bacterial load means (log₁₀ CFU/g) in spleen, lung and liver in mice after 24 h of non-treated and cefiderocol treated groups; along with the cefiderocol efficacy, measured as the net bacterial load reductions (Vehicle - FDC treatments), and differences in bacterial load reduction between pre-treatment and post-treatment isolates. Statistical analysis was performed by two-way ANOVA with Tukey's multiple comparisons test.

| ***P. aeruginosa* isolates** | **Spleen** | | | | |  | **Lung** | | | | |  | **Liver** | | | | |
| --- | --- | --- | --- | --- | --- | --- | --- | --- | --- | --- | --- | --- | --- | --- | --- | --- | --- |
|  | **Vehicle** | **FDC** | **Vehicle - FDC (FDC efficacy)** | **Difference between isolates** | **Statistical analysis/P value** |  | **Vehicle** | **FDC** | **Vehicle - FDC (FDC efficacy)** | **Difference between isolates** | **Statistical analysis/P value** |  | **Vehicle** | **FDC** | **Vehicle - FDC (FDC efficacy)** | **Difference between isolates** | **Statistical analysis/P value** |
| **101-E5**  **(pre-treatment)** | 8.74 | 7.07 | 1.67 | 0.56 | 0.0849 |  | 8.63 | 6.49 | 2.14 | 1.17 | 0.0251  (*) |  | 7.99 | 5.05 | 2.94 | 2.06 | <0.001  (**) |
| **103-H8**  **(post-treatment)** | 8.77 | 7.66 | 1.11 |  |  |  | 8.48 | 7.51 | 0.97 |  |  |  | 7.91 | 7.03 | 0.88 |  |  |
| **5A-HUSE**  **(pre-treatment)** | 8.53 | 6.98 | 1.55 | 0.77 | 0.0022 (**) |  | 7.36 | 5.52 | 1.84 | 0.87 | <0.001  (**) |  | 8.02 | 6.17 | 1.85 | 0.46 | 0.0188  (*) |
| **5B-HUSE**  **(post-treatment)** | 8.59 | 7.81 | 0.78 |  |  |  | 7.6 | 6.64 | 0.97 |  |  |  | 8.15 | 6.76 | 1.39 |  |  |
| **11-CHUAC**  **(post-treatment)** | 9.26 | 8.42 | 0.84 | NA | NA |  | 8.63 | 7.8 | 0.83 | NA | NA |  | 8.16 | 7.57 | 0.59 | NA | NA |

**P* < 0.05, ***P* < 0.01. NA, not adequate.

**Supplementary Table 2:** Bacterial inocula used in murine sepsis models.

| ***P. aeruginosa* isolate ID** | | **Inoculum concentration (CFU/mL)** | **Bacterial challenge (CFU/mouse) *** | **Baseline bacterial burden, 2 hours post-infection (CFU/gr of lung)** |
| --- | --- | --- | --- | --- |
| **101-E5** | | 7.5 x 10^8^ | 7.5 x 10^7^ | 1.2 × 10^7^ |
|  | **103-H8** | 7.1 x 10^8^ | 7.1 x 10^7^ | 6.3 × 10^6^ |
|  | **5A-HUSE** | 3.3 x 10^9^ | 3.3 x 10^8^ | 7.7 × 10^6^ |
|  | **5B-HUSE** | 3.1 x 10^9^ | 3.1 x 10^8^ | 6.0 × 10^6^ |
|  | **11-CHUAC** | 8.4 x 10^8^ | 8.4 x 10^7^ | **2.8 × 10^7^** |

*100 µl were inoculated in each mouse.

**SUPPLEMENTARY MATERIAL AND METHODS**

**Antimicrobial susceptibility testing**

The MICs of ceftazidime, ceftazidime/avibactam, ceftolozane/tazobactam, imipenem, imipenem/relebactam and cefiderocol were determined in triplicate, by broth microdilution assays. All assays were performed using cation-adjusted Mueller-Hinton broth (CAMHB), except for cefiderocol, which was determined using iron-depleted CAMHB prepared according to CLSI M100 guidelines. EUCAST v14.0 clinical breakpoints and guidelines (http://www.eucast.org/clinical_breakpoints/) were used for reference purposes. *E. coli* ATCC 25922 and *P. aeruginosa* ATCC 27853 strains were used as controls.
